# PEO-Based Block Copolymer Electrolytes Containing Double Conductive Phases with Improved Mechanical and Electrochemical Properties

**DOI:** 10.3390/ma15227930

**Published:** 2022-11-09

**Authors:** Ze-Kun Zhang, Shi-Peng Ding, Ze Ye, Ding-Li Xia, Jun-Ting Xu

**Affiliations:** State Key Laboratory of Motor Vehicle Biofuel Technology, International Research Center for X Polymers, Department of Polymer Science and Engineering, Zhejiang University, Hangzhou 310027, China

**Keywords:** block copolymer, solid electrolytes, double conductive phases, mechanical strength, electrochemical properties

## Abstract

In this work, the advanced all solid-state block copolymer electrolytes (SBCPEs) for lithium-ion batteries with double conductive phases, poly(ethylene oxide)-*b*-poly(trimethyl-*N*-((2-(dimethylamino)ethyl methacrylate)-7-propyl)-ammonium bis(trifluoromethanesulfonyl) imide) (PEO-*b*-PDM-dTFSI)/LiTFSI, were fabricated, in which the charged PDM-dTFSI block contained double quaternary ammonium cations and the PEO block was doped with LiTFSI. The disordered (DIS) and ordered lamellae (LAM) phase structures were achieved by adjusting the composition of the block copolymer and the doping ratio *r*. In addition, the presence of the hard PDM-dTFSI block and the formation of the LAM phase structure resulted in a good mechanical strength of the solid PEO-*b*-PDM-dTFSI/LiTFSI electrolyte, and it could maintain a high level of 10^4^ Pa at 100 °C, which was around 10,000 times stronger than that of the PEO/LiTFSI electrolyte. Based on the good mechanical and electrochemical properties, the PEO-*b*-PDM-dTFSI/LiTFSI SBCPE exhibited excellent long-term galvanostatic cycle performance, indicating the strong ability to suppress lithium dendrites.

## 1. Introduction

Lithium-ion batteries (LIBs) play an important role in modern electronic devices and vehicles, and new generation batteries providing a higher energy density need to be developed to fulfill the increasing market demand [1,2,3]. Lithium metal is regarded as an excellent anode candidate to offer high energy density, but it will cause some serious problems when matched with flammable liquid electrolytes such as electrolyte leakage, fire, and explosion [4,5]. The solid state electrolyte may suppress the growth of lithium dendrite to improve the safety of LIBs; in particular, polymer electrolytes have received great attention due to its chemical stability and flexibility [6,7,8].

The poly(ethylene oxide) (PEO)/salt hybrid is a representative polymer electrolyte due to the effective dissociation of lithium salts and the good mobility of the PEO segment [9,10,11]. Nevertheless, the crystallization of PEO usually results in low ionic conductivity at room temperature [12,13], and the soft PEO chains cannot offer sufficient mechanical strength to inhibit the penetration of lithium dendrites [14]. To solve this problem, solid-state block copolymer (BCP) electrolytes (SBCPEs) comprising the stiff blocks have been developed. BCP electrolytes can form various nanostructures in bulk via microphase separation such as lamellae (LAM), cylinders, spheres. and etc. [15,16], which will construct the lithium ion conducting channels and the stiff domains [17,18,19,20]. For example, the frequently reported salt-doped PEO-*b*-poly(styrene) (PEO-*b*-PS) BCPs could provide a good mechanical strength and ionic conductivity (*σ*), but the *σ* value is usually lower than that of the pure PEO/salt electrolytes [21,22,23,24]. Additionally, many PEO-based BCPs with single-ion blocks have been developed to improve the electrochemical performance of SBCPEs [25], that is, the anions were covalently attached on the polymer chains and only the Li^+^ could migrate. Indeed, this greatly enhanced the lithium ion transfer number (*t*_Li_^+^), however, a decreased ionic conductivity was usually encountered due to the slow motion of the single-ion polymer chains [26,27,28]. The ionic liquid (IL) is the good additive for BCP electrolytes to improve the *σ* and electrochemical stability [29,30,31,32]. Fan and Shen fabricated a series of mesogen-jacketed liquid crystalline (MJLC) BCP/lithium salt/IL ternary hybrids [33], which could maintain high ionic conductivities and storage moduli at high temperatures, while the plasticization effect of ILs on other common BCPs will be more profound compared with the rigid MJLC BCPs and will sacrifice the mechanical strength of the electrolytes [34]. Therefore, simultaneous improvement of the ionic conductivity, mechanical strength, and electrochemical performances of the SBCPEs is always a contradictory challenge.

To find a good strategy to balance the different properties of the SBCPEs, BCPs with double conductive phases were proposed in our previous work. Both the soft and rigid blocks of these BCPs can associate with the lithium ions, and the phase morphologies and properties are dependent on the salt doping ratio (*r*) [35,36,37]. It was found that the ionic conductivity and the mechanical strength were simultaneously improved in the poly(propylene monothiocarbonate)-*b*-poly(ethylene oxide) (PPMTC-*b*-PEO)/LiTFSI hybrids [38], which can be mainly attributed to the presence of the second conductive phase and the microphase-separated structure. However, at a higher doping ratio (*r* = 1/3), the storage modulus of the PEO-*b*-PPMTC/LiTFSI hybrid was seriously decreased, arising from the plasticization of LiTFSI, though the *σ* reached a high level of 10^−4^ S/cm at 30 °C [38]; moreover, the measurement of the electrochemical performance was hard to conduct on these viscous electrolytes with a high *r* because of the failure of film formation, which limits their further practical application. Therefore, it is necessary to find a better conductive rigid block for the PEO-based SBCPE to meet the test requirement of the LIBs and improve their electrochemical performances.

In recent years, the studies on the poly(ionic liquid)s (PILs)-based electrolytes have attracted much attention due to its extensive synthetic strategies [39] and excellent electrochemical stability [40]. On the other hand, the introduction of PIL blocks into BCPs can improve the phase separation strength of BCPs and induce the formation of ordered ion-conducting channels [41,42]. In PIL/salt hybrids, the transportation of Li^+^ is mainly dominated by the “vehicular mechanism”, and the polycations can also interact with the anions from lithium salts [7,43]. In this case, the lithium ion transfer number and electrochemical stability of the PIL/salt electrolytes were improved [44,45], and the *t*_Li_^+^ was higher for the PILs with multiple cations [46]. With the consideration of the excellent electrochemical stability of PILs and the good conductivity and mechanical strength of double conductive phases, in this work, the novel poly(trimethyl-*N*-((2-(dimethylamino)ethyl methacrylate)-7-propyl)-ammonium bis(trifluoromethanesulfonyl) imide) (PDM-dTFSI) block with double quaternary ammonium cations was introduced into the PEO-*b*-PDM-dTFSI BCPs as one of the conductive phases for the first time. The relative rigid PDM-dTFSI block can not only provide mechanical strength but also contribute to ionic conductivity as a second conductive phase, which is expected to simultaneously improve the mechanical strength and electrochemical performances of the SBCPEs. The effects of the BCP composition and salt doping ratio on the phase-separated structures and ionic conductivities of the PEO-*b*-PDM-dTFSI/LiTFSI SBCPEs were systematically investigated. The SBCPE with the optimal ionic conductivity was chosen to proceed with further electrochemical measurements, demonstrating the good film-forming capacity and stable long-term lithium stripping and plating performance. This work provides an efficient idea to trade-off the ionic conductivity, mechanical strength, and electrochemical performance of SBCPEs.

## 2. Materials and Methods

### 2.1. Materials

2-(Dimethylamino)ethyl methacrylate (DM, 99%), 2-bromoisobutyryl bromide (98%), and 1,1,4,7,10,10-hexamethyltriethylenetetramine (HMTTA) were purchased from Aladdin. Polyethylene glycol monomethyl ether (PEO, *M*_n_ = 5000 g/mol) and (3-bromopropyl)trimethylammonium bromide (97%) were obtained from TCI. PEO and DM monomers were washed with basic alumina to remove the polymerization inhibitors prior to use. Tetrahydrofuran (THF) from Sinopharm Chemicals was refluxed with sodium hydride. Super dry dimethylformamide (DMF) and bis(trifluoromethane) sulfonimide lithium (LiTFSI, 98%) from J&K Scientific were used as received. Li metal anode (diameter = 12 mm) was obtained from China Energy Lithium Co., Ltd., Tianjin, China.

### 2.2. Synthesis of PEO-b-PDM BCPs

PEO-*b*-PDM BCPs were synthesized by atom transfer radical polymerization (ATRP). First, the PEO-Br macroinitiator was prepared by esterification of PEO with 2-bromoisobutyryl bromide. Then, a series of PEO-*b*-PDM*_n_* BCPs were obtained through conducting the ATRP polymerization of DM, where the subscription *n* represents the polymerization degree of the PDM block. The specific experimental process and characterization results of PEO-*b*-PDM BCPs can be found in the Supplementary Materials.

### 2.3. Synthesis of PEO-b-PDM-dTFSI Charged BCPs

**Scheme 1:**
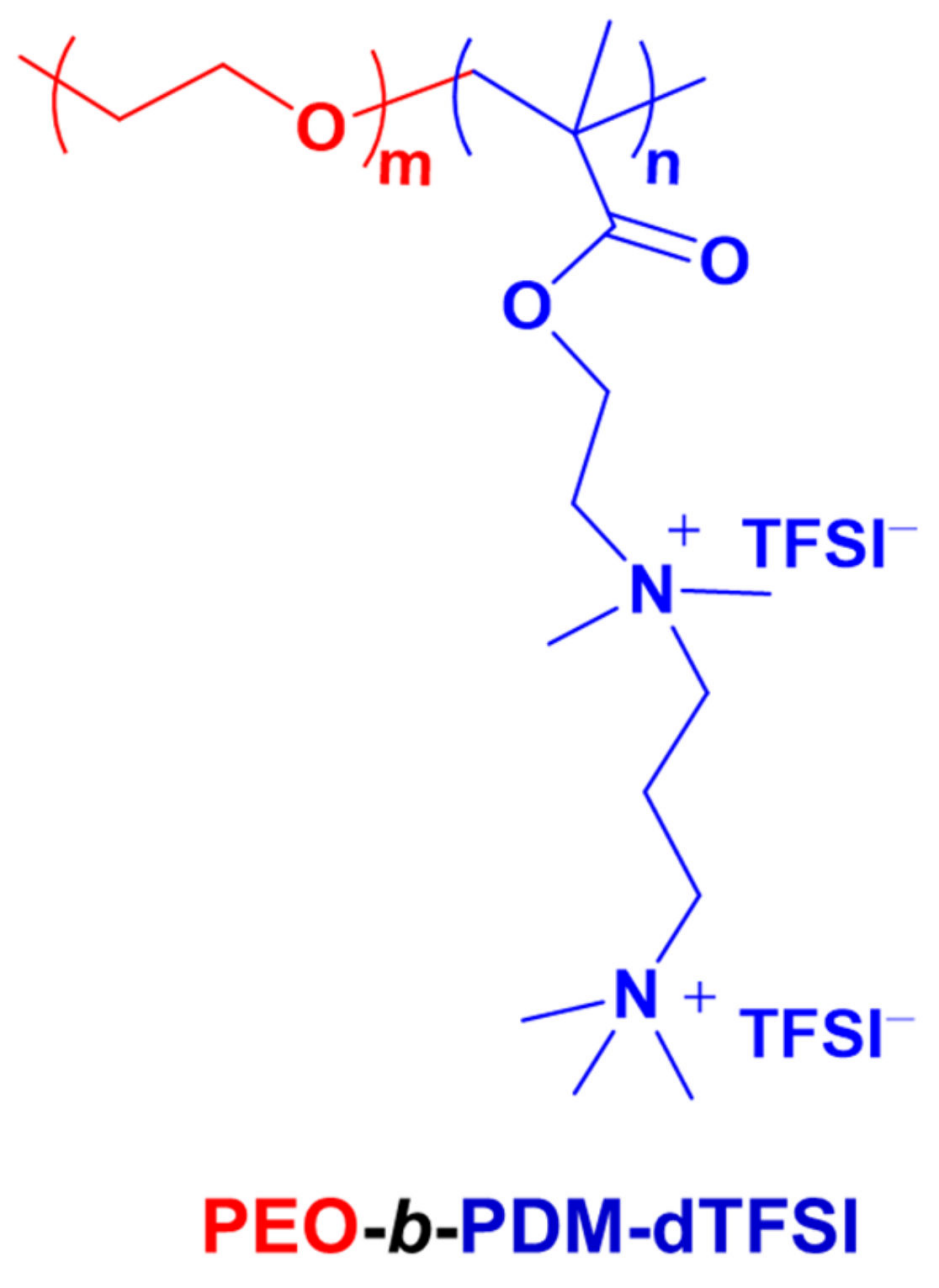
Structure of the PEO-*b*-PDM-dTFSI BCP.

First, the PDM block of PEO-*b*-PDM*_n_* BCPs was quaternized with (3-bromopropyl)trimethylammonium bromide to give the PEO-*b*-PDM*_n_*-dBr intermediate, then the target products of PEO-*b*-PDM*_n_*-dTFSI BCPs were obtained by the ionic exchange reaction of PEO-*b*-PDM*_n_*-dBr against LiTFSI in DMF. The detailed process is described in the Supplementary Materials, and the structure and the molecular characteristics of PEO-*b*-PDM-dTFSI BCP are illustrated in Scheme 1 and Table 1, respectively.

### 2.4. Fabrication of Solid-State Block Copolymer Electrolytes

**Scheme 2:**
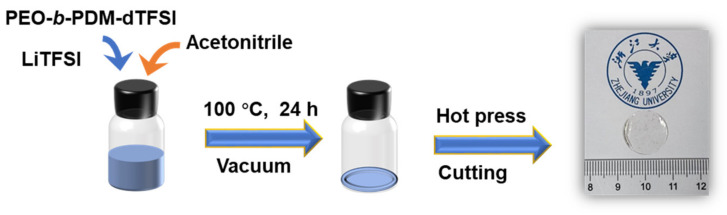
Schematic illustration of the fabrication of SBCPEs.

A certain amount of acetonitrile solution of PEO-*b*-PDM*_n_* and LiTFSI salt was mixed and stirred for 12 h to blend homogeneously. The above solution was first placed under dynamic vacuum for 8 h at room temperature and then evacuated in a vacuum oven at 100 °C for 24 h to completely remove the solvent. Finally, the obtained solid electrolytes were stored in the glove box filled with nitrogen (the content of O_2_ and H_2_O is less than 0.1 ppm) and named as PEO-*b*-PDM*_n_*-dTFSI-*r*, where *r* is the molar ratio of LiTFSI to the sum of EO units and N cation (i.e., [Li^+^]/([EO]+[N^+^])). The solid electrolyte used for the assembling cell was hot-pressed into a film with a thickness of 100 μm at 40 °C and then cut into a circular film with a diameter of 16 mm (Scheme 2). 

### 2.5. Battery Assembly

All cells including Li/Li symmetric cells and Li/stainless steel (SS) cells were assembled in the glove box filled with argon. The edges of the electrolyte will become slightly thicker after being cut into round films, which may result in the non-uniform contact between the electrolyte film and the electrode. Therefore, before testing, the cells were heated under 60 °C for 24 h to achieve good contact between the electrodes and the solid polymer electrolyte.

### 2.6. Characterizations

The composition and number-averaged molecular weight (*M*_n_) of the samples were calculated according to the ^1^H-NMR spectra recorded on a Bruker DMX-400 instrument (400 MHz). The polydispersity indices of PEO-*b*-PDM*_n_* BCPs were measured by gel permeation chromatography (GPC) on a Waters system instrument using DMF containing 0.05 M LiBr as the mobile phase with a leaching rate of 1 mL/min at 40 °C, calibrated by the polystyrene standards. Differential scanning calorimetry (DSC) characterization was conducted on a TA DSC 25 instrument. About 5 mg of the sample was sealed in an aluminum pan, and the first cooling and second heating curves at the ramp rates of 20 °C/min were plotted. Small-angle X-ray scattering (SAXS) measurement was performed on the BL16B1 beamline of the Shanghai Synchrotron Radiation Facility (SSRF) equipped with a Pilatus 2M detector. The energy of the incident X-ray was 10 keV and the distance from the sample to detector was 1940 mm. All of the samples were annealed at 100 °C under dynamic vacuum for 24 h and then naturally cooled down to room temperature. The samples for temperature-variable tests were heated from 30 to 100 °C at a rate of 10 °C/min. Before data collection, the sample was held at the target temperature for about 3 min to achieve thermal equilibrium. The raw two-dimensional patterns were transformed into one-dimensional data using the Fit 2D tool, and the scattering vector (*q*) of the SAXS profiles was calibrated with silver behenate. Rheological tests were carried out on a HAAKE MARS 60 rheometer using 20 mm diameter parallel plates, and the gap thickness was 1 mm. A fixed strain amplitude (1%) was used, and the temperature sweep data with a frequency of 1 Hz between 30 and 100 °C were collected upon heating with a heating rate of 2 °C/min. During the tests, the polymer electrolyte may inevitably absorb trace amounts of water, resulting in slightly lower mechanical strength than the true value. The temperature-variable ionic conductivity (*σ*) of the solid polymer electrolyte film was measured on a CHI660E electrochemical workstation equipped with a Linkam hot stage at a frequency range from 1 MHz to 1 Hz. The electrolyte films were sandwiched between two stainless steel electrodes. The bulk resistance (*R*) was determined by extrapolating the high-frequency plateau value of the real part impedance. The *σ* was obtained via the equation *σ* = *L*/(*SR*), where *L* and *S* are the thickness and area of the electrolyte film, respectively. The thicknesses of the electrolytes used for the conductivity measurements are 1 mm. The average conductivity was taken from the three parallel tests. The lithium ion transference number (*t*_Li_^+^) was obtained on the CHI660E electrochemical workstation at 30 °C by assembling Li/Li symmetric cells. *t*_Li_^+^ is calculated by the equation: *t*_Li_^+^ = [*I*_s_(Δ*V* − *I*_0_*R*_0_)]/[*I*_0_(Δ*V* − *I*_s_*R*_s_), where Δ*V* is the applied polarization voltage of 10 mV, *I*_0_ and *I*_s_ are the initial and the steady-state current measured by DC polarization, respectively. *R*_0_ and *R*_s_ represent the interfacial resistance before and after polarization obtained from the AC impedance spectrum, respectively. Linear sweep voltammetry (LSV) was also conducted on the CHI660E electrochemical workstation. The Li/SS cell with stainless steel as the working electrode and Li metal as the counter electrode was assembled. The cut-off voltage and sweep rate were 6 V and 1 mV/s, respectively. The constant current cycling performance of the Li/Li symmetrical cells was performed on the LAND device with the current densities of 0.05 mA/cm^2^ and 0.1 mA/cm^2^ at 60 °C.

## 3. Results and Discussion

### 3.1. Thermal and Phase Behavior of the PEO-b-PDM-dTFSI/LiTFSI Electrolytes

**Figure 1:**
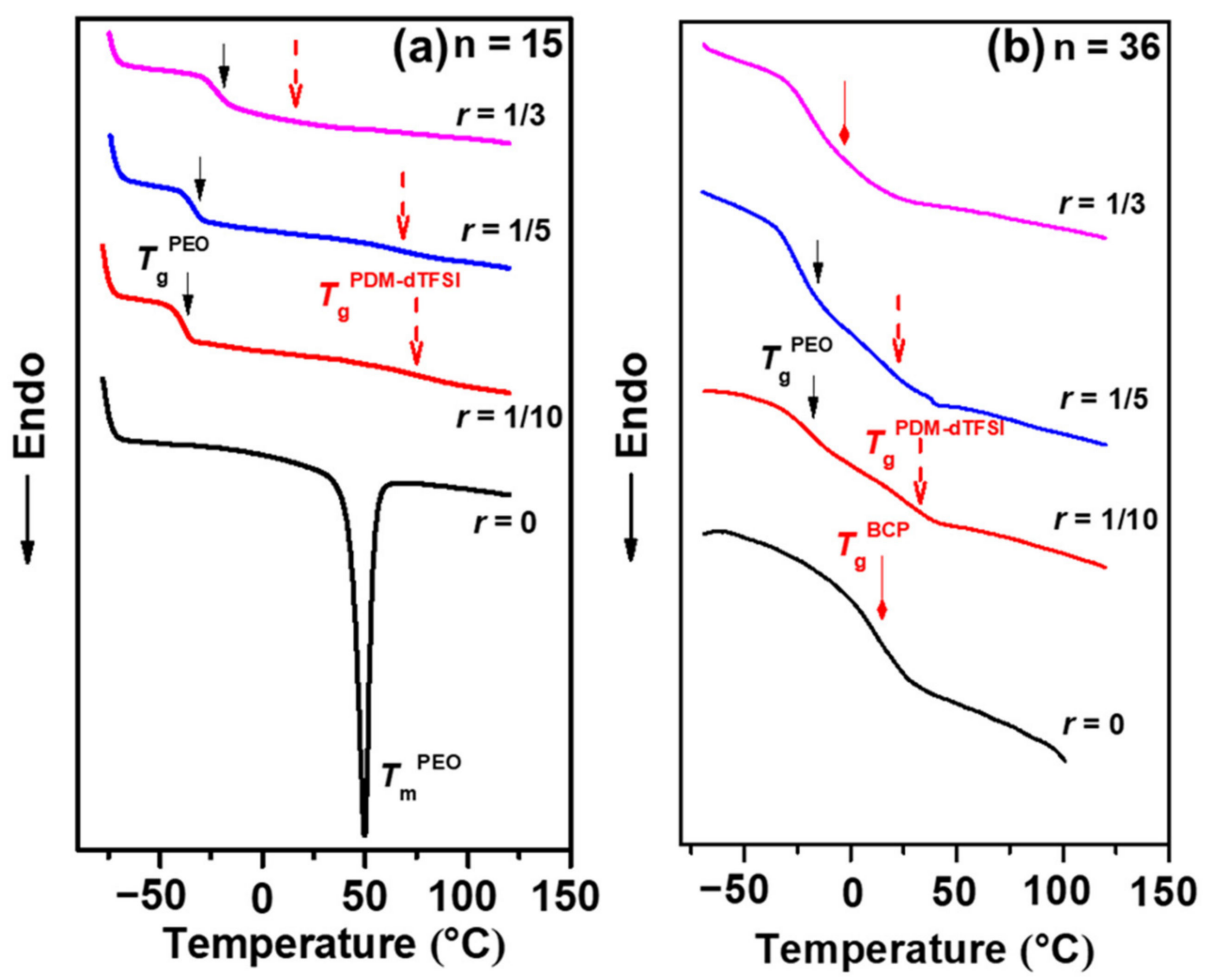
The DSC curves of the PEO_114_-*b*-PDM*_n_*-dTFSI/LiTFSI electrolytes when *n* is (**a**) 15 and (**b**) 36 in the second heating scans.

The crystallization behavior and glass transition temperature (*T*_g_) of the PEO_114_-*b*-PDM*_n_*-dTFSI/LiTFSI hybrids were determined by DSC. As shown in Figure 1a, a distinct endothermic peak was observed in PEO_114_-*b*-PDM_15_-dTFSI, representing the melting peak of the PEO block (*T*_m_ = 49.7 °C), and this peak disappeared in the salt-doped PEO_114_-*b*-PDM_15_ hybrids, which can be attributed to the suppression of the PEO crystallization after the lithium ions complex with the PEO chains. In addition, all the PEO_114_-*b*-PDM_15_-dTFSI/LiTFSI hybrids exhibited two glass transition steps, indicating the occurrence of the phase separation between these two blocks. For instance, the double *T*_g_s of the PEO_114_-*b*-PDM_15_-dTFSI-1/10 hybrids were assigned to the PEO (*T*_g_^PEO^ = −36.8 °C) and PDM-dTFSI (*T*_g_^PDM-dTFSI^ = 74.2 °C) blocks, respectively. With the increase in the doping ratio *r*, the *T*_g_^PEO^ increased while *T*_g_^PDM-dTFSI^ decreased (Figure 1a and Table 2). The increased *T*_g_^PEO^ was ascribed to the higher number of associating points between the Li^+^ and EO units, lowering the mobility of PEO segments [47]. In contrast, the gradual decrease in *T*_g_^PDM-dTFSI^ resulted from a disruption of the aggregation between the charged PDM-dTFSI chains and the TFSI^−^ in the presence of LiTFSI. The significant drop in *T*_g_ with the increase in the salt content has also been reported in the PIL/salt hybrids [44]. When the polymerization degree of the PDM-dTFSI block is increased to 36, one can see only one *T*_g_ at ~11.5 °C for the salt-absent sample. However, the DSC curves of the PEO_114_-*b*-PDM_36_-dTFSI-*r* hybrids contained two separated *T*_g_s at *r* = 1/10 and 1/5, and then re-back to only one *T*_g_ at a higher doping ratio (*r* = 1/3), reflecting the complicated change in the phase separation state with *r* in PEO_114_-*b*-PDM_36_-dTFSI/LiTFSI hybrids. Therefore, the obtained PEO_114_-*b*-PDM*_n_*-dTFSI-*r* electrolytes possessed the soft (PEO) and relatively hard (PDM-dTFSI) block, and the crystallization of the PEO block could be fully suppressed by adjusting the composition and the doping ratio. On the other hand, the phase separation behavior of the salt-doped PEO_114_-*b*-PDM*_n_*-dTFSI was considered to be relevant with the salt content, which will be confirmed by the SAXS results.

The phase behavior of the PEO-*b*-PDM-dTFSI BCPs was studied first. The SAXS profiles of the PEO_114_-*b*-PDM*_n_*-dTFSI BCPs with different PDM-dTFSI block lengths are shown in Figure S5. The scattering peaks with the vector ratio of 1:2 was observed when *n* = 15, implying the formation of a LAM structure. When the PDM-dTFSI content increased (*n* = 36 and 55), the LAM structure disappeared and was replaced by the compatible state, as revealed by the absence of the primary scattering peaks (Figure S5). This is because the *f*_PEO_ is decreased with the increase in the polymerization degree of the PDM-dTFSI block (Table 1), which will decrease the phase separation ability between two blocks.

**Figure 2:**
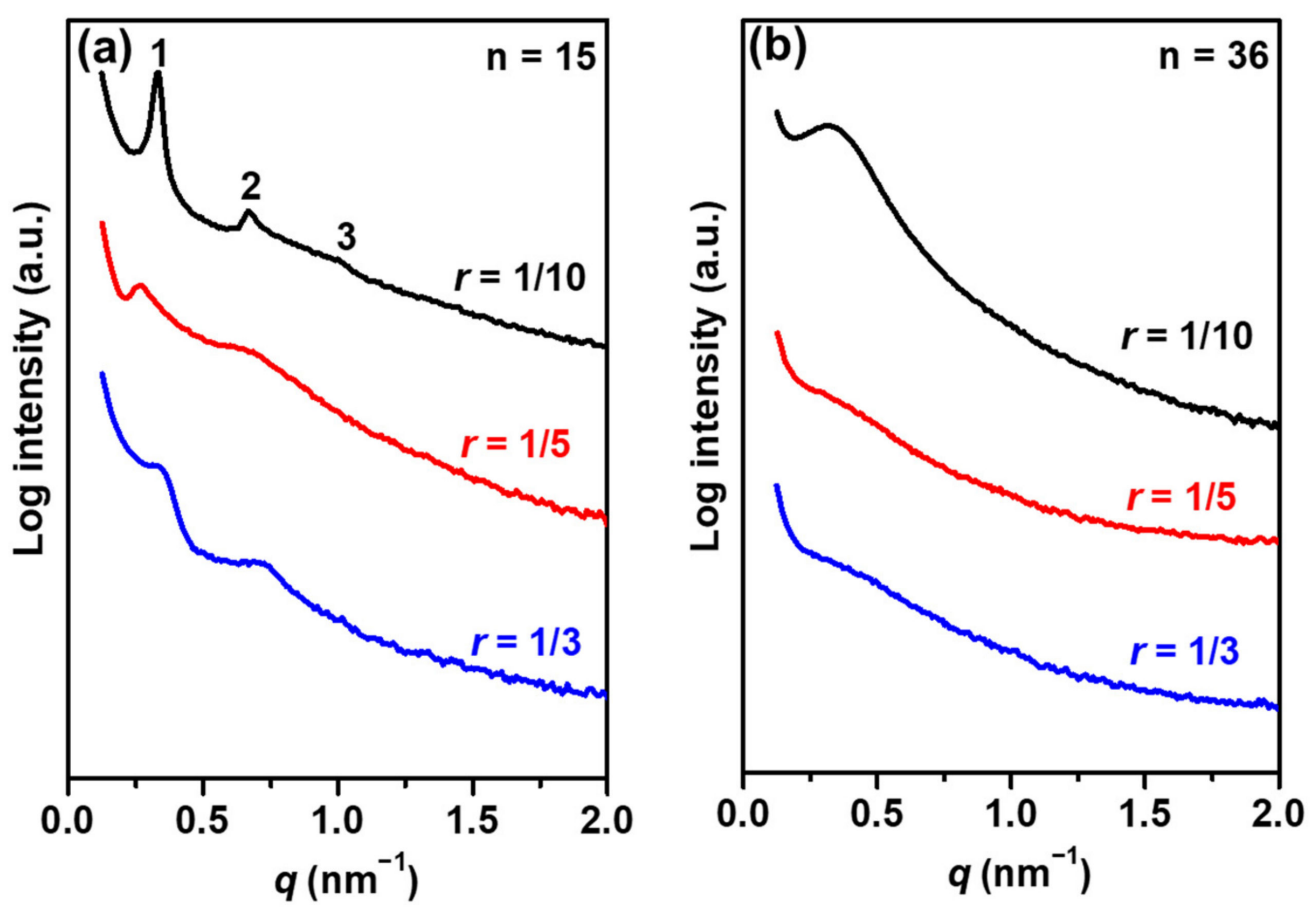
SAXS profiles of the PEO_114_-*b*-PDM*_n_*-dTFSI/LiTFSI electrolytes with different doping ratios at 30 °C when (**a**) *n* =15 and (**b**) 36.

After doping with salts, the phase behavior of the PEO-*b*-PDM-dTFSI/LiTFSI electrolytes was different from that of the undoped BCPs, which varied with the doping ratio *r*. As shown in Figure 2a, the ordered LAM structure remained in the PEO_114_-*b*-PDM_15_-dTFSI/LiTFSI hybrid with *r* = 1/10, while the phase structure became less ordered with the increasing doping ratio, as confirmed by the broader first-order peaks at *r* = 1/5 and 1/3 (Figure 2a). As for the PEO_114_-*b*-PDM_36_-dTFSI/LiTFSI electrolytes, only one primary peak could be observed when the salt ratio was 1/10, indicating the occurrence of the disordered phase structure induced by salt doping (Figure 2b). In contrast, the scattering peaks were unobvious and almost disappeared when *r* was increased to 1/5 and 1/3, implying that the phase separation tendency was weakened at higher doping ratios, which was similar to that of the PEO_114_-*b*-PDM_15_-dTFSI/LiTFSI hybrids. The increased compatibility between two blocks was also in accordance with the DSC results (Figure 1b), where the glass transitions of two phases can hardly be distinguished at *r* = 1/3. In the PEO-*b*-PDM-dTFSI/LiTFSI electrolyte, the LiTFSI can be dissolved into the PEO phase due to the complexation between the Li^+^ and -C-O-C- group of the PEO chains [48]. Meanwhile, the quaternary ammonium cations and TFSI anions of the charged PDM-dTFSI block had the ability to electrostatically associate with the TFSI^−^ and Li^+^, respectively [43], so that the lithium salts also existed in the PDM-dTFSI domain, especially at high salt contents. When salts were located in both phases, the electrostatic interaction between these two blocks was resultantly produced, and the attraction will be increased and become more remarkable with the increase in *r*. This is why the enhanced compatibility between two blocks was observed at higher doping ratios (Figure 2). The above results indicate that the ordered LAM or disordered structure can be achieved in PEO_114_-*b*-PDM*_n_*-dTFSI/LiTFSI electrolytes by adjusting the length of the charged block and the doping ratio *r*. Since both phases carry charges, double ion-channels are provided for the transport of lithium ions. On the other hand, the ordered LAM structure endows the solid electrolyte film with good mechanical properties, which will be examined in the following part.

### 3.2. Mechanical Properties of the PEO-b-PDM-dTFSI/LiTFSI Electrolytes

**Figure 3:**
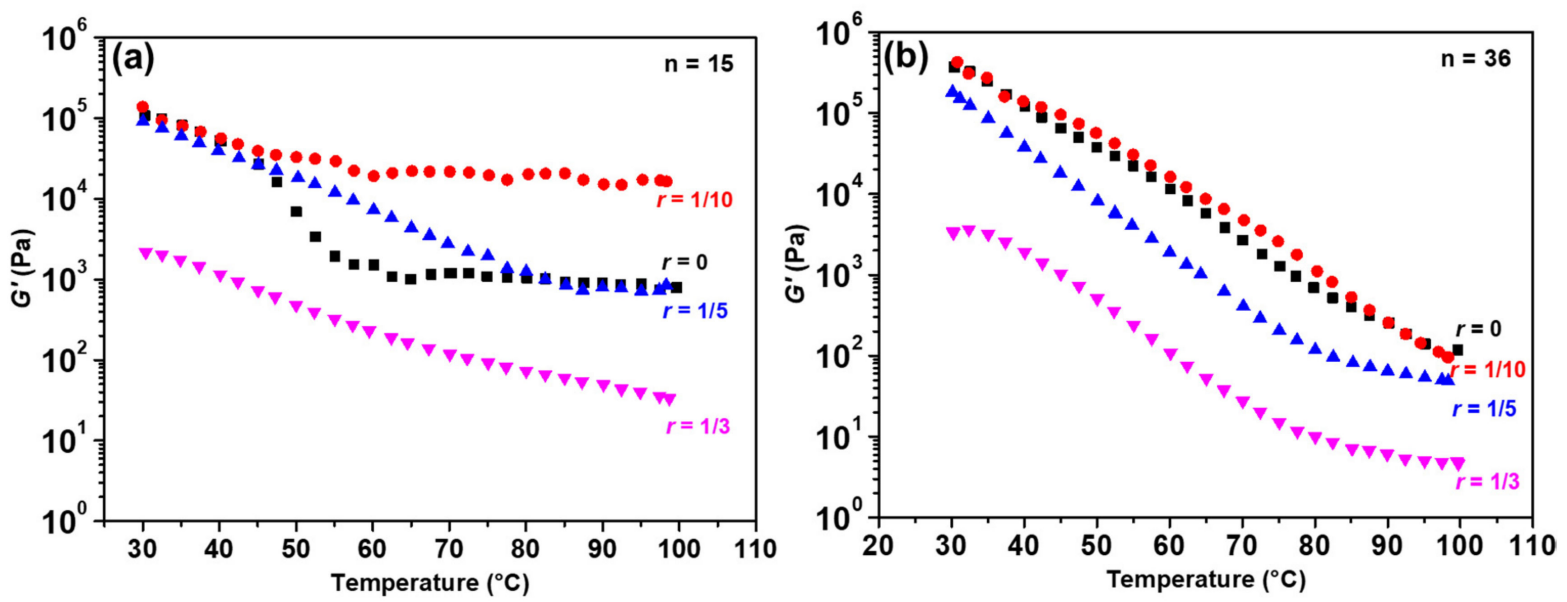
Storage moduli (*G’*) of the PEO_114_-*b*-PDM*_n_*-dTFSI/LiTFSI electrolytes with different doping ratios upon heating when (**a**) *n* =15 and (**b**) 36.

The mechanical properties of the solid electrolyte films were investigated by the temperature-variable rheological tests. As for the PEO_114_-*b*-PDM_15_-dTFSI/LiTFSI electrolytes (Figure 3a), the storage modulus (*G’*) of the salt-absent sample was around 10^5^ Pa at 30 °C, but suddenly dropped to 10^3^ Pa during 45–60 °C due to melting of the PEO block (Figure 1a). When the salt doping ratio *r* was increased to 1/10 and 1/5, the *G’* maintained the level of 10^5^ Pa at room temperature and changed moderately with the increase in temperature, and was still higher than 10^4^ and 10^3^ Pa at 100 °C, respectively. Specifically, the mechanical properties of the electrolyte at *r* = 1/10 exhibited an excellent temperature stability (Figure 3a). This is because the high ordered and thermally stable LAM structure is formed in the PEO_114_-*b*-PDM_15_-dTFSI-1/10 hybrid (Figure S6). It should be noted that the mechanical properties of the PEO_114_-*b*-PDM_15_-dTFSI/LiTFSI hybrids was far better than that of the typical PEO/salt electrolytes (below10 Pa at the molten state) [38], which will enhance the ability to resist lithium dendrite and improve the cycle performance of the lithium-ion battery. In order to confirm the influence of the phase separation on the mechanical strength of the BCP electrolyte, the moduli of the PEO_114_-*b*-PDM_36_-dTFSI/LiTFSI hybrids were further studied. Theoretically, the *G’* of the PEO_114_-*b*-PDM*_n_*-dTFSI/LiTFSI with *n* = 36 should be better than that of *n* = 15 due to its higher content of the rigid PDM-dTFSI block. Nevertheless, the temperature-variable rheological experiment showed that the storage moduli of the PEO_114_-*b*-PDM_36_-dTFSI/LiTFSI hybrids decreased rapidly with the increase in temperature and was only ~100 Pa at 100 °C when *r* ≤ 1/5 (Figure 3b). The relatively poor mechanical property can be attributed to the weak and disordered phase structure of the PEO_114_-*b*-PDM_36_-dTFSI/LiTFSI BCPs (Figure 2b). In our previous studies, the mechanical strength improvements were also achieved in the phase separated BCP electrolyte compared to the BCPs with a homogenous state [38]. At the high doping ratio (*r* = 1/3), both the PEO_114_-*b*-PDM*_n_*-dTFSI/LiTFSI (*n* = 36 and 15) electrolytes exhibited lower moduli than the hybrids with *r* = 1/10 and *r* = 1/5 (Figure 3), which is probably due to the plasticization of lithium salts to the BCPs together with the weaker phase separation ability of the electrolyte at the high salt content.

### 3.3. Electrochemical Properties of the PEO-b-PDM-dTFSI/LiTFSI Electrolytes

**Figure 4:**
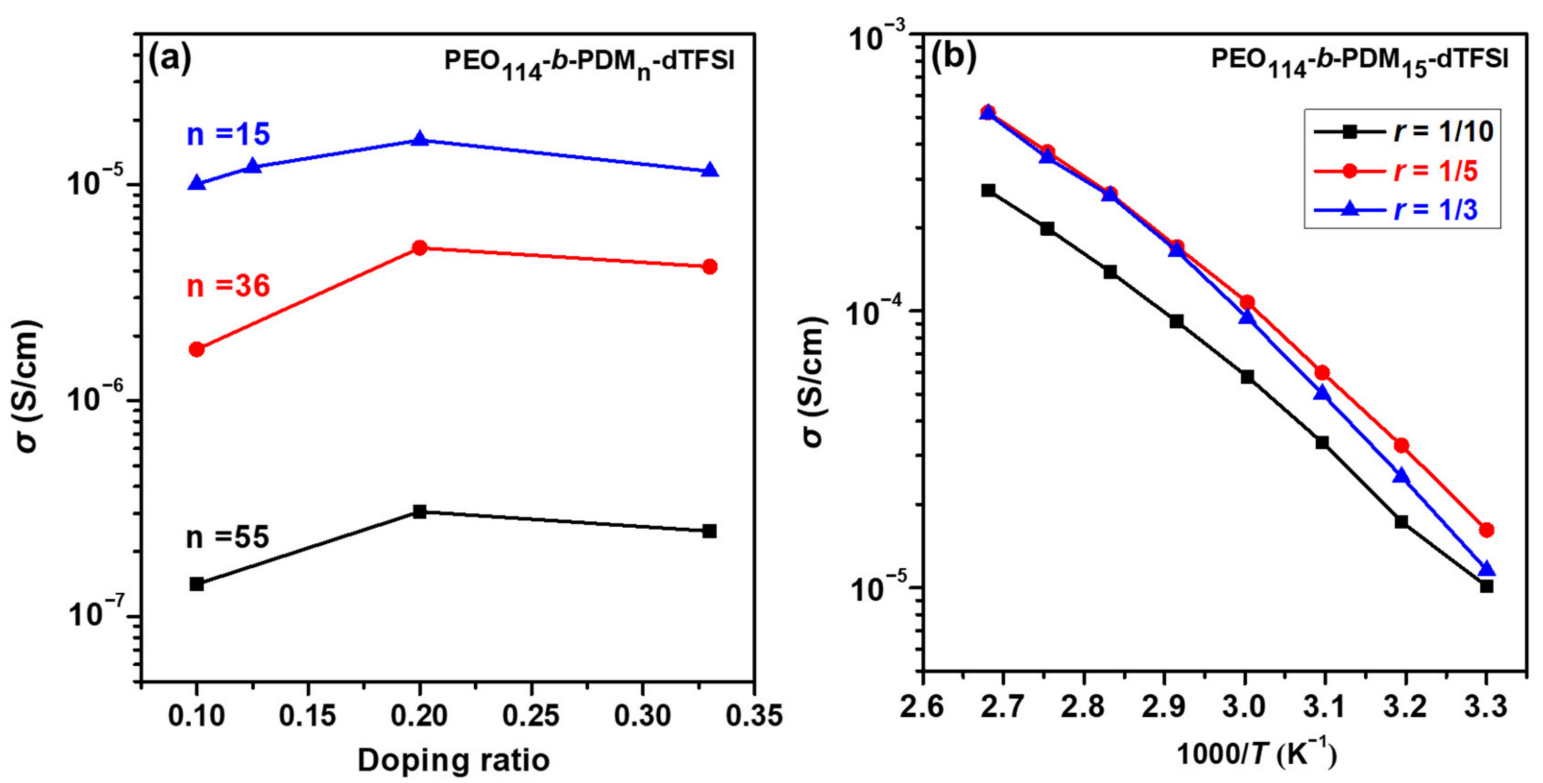
(**a**) Ionic conductivity of the PEO_114_-*b*-PDM*_n_*-dTFSI/LiTFSI hybrids at 30 °C. (**b**) Temperature dependence of ionic conductivity of PEO_114_-*b*-PDM_15_-dTFSI/LiTFSI hybrids with different doping ratios.

First, the ionic conductivity of the PEO_114_-*b*-PDM*_n_*-dTFSI/LiTFSI hybrids with various *r*_s_ was studied. As shown in Figure 4a, the similar changing trend of ionic conductivity was observed for all three samples with different PDM-dTFSI block lengths, that is, the ionic conductivity increased first and then decreased as the doping ratio *r* rose. On the other hand, it was found that the PEO_114_-*b*-PDM_15_-dTFSI sample possessed the highest ionic conductivity among these three BCPs, and the doping ratio for achieving its optimal *σ* in our experimental doping range was 1/5. In the commonly studied PEO/LiTFSI and PEO-*b*-PS/LiTFSI electrolytes, the *r* was ~1/12 when the conductivity was maximized [49], which was much lower than that of the PEO_114_-*b*-PDM*_n_*-dTFSI BCPs. The PDM-dTFSI blocks with double cationic groups could also dissolve the lithium salts, which will alleviate the aggregation of lithium salts and increase the number of “free” charge carriers. Therefore, the PEO_114_-*b*-PDM*_n_*-dTFSI BCPs achieved the maximal *σ* at a larger *r*. A similar situation has also been reported in some PEO-*b*-poly(zwitterion)/salt hybrids [50]. On the other hand, it was found that the ionic conductivity of the electrolytes increased with the decreasing *n*, and the highest *σ* reached the order of 10^−5^ S/cm in the PEO_114_-*b*-PDM_15_-dTFSI-1/5 hybrid at 30 °C. As for the PEO_114_-*b*-PDM*_n_*-dTFSI/LiTFSI hybrids, the PEO block was the major conductive phase, which is mainly responsible for transporting ions, so a higher *σ* value was obtained in the BCPs with a larger volume fraction of the PEO block (Table 1). Furthermore, the temperature-variable conductivity was measured for the PEO_114_-*b*-PDM_15_-dTFSI/LiTFSI hybrids at *r* = 1/10, 1/5, and 1/3. It can be seen from Figure 4b that the ionic conductivity at different *r*s increased with a temperature rise in the range of 30–100 °C, and the *σ* values at the optimal ratio (*r* = 1/5) were 1.6 × 10^−5^ and 1.1 × 10^−4^ S/cm at 30 °C and 60 °C, respectively, which are comparable to most solid polymer electrolytes [51,52,53] and are able to meet the requirement for lithium ion cells. In addition, the conductivity–temperature curve was fitted using the Vogel-Tammann-Fulcher (VTF) equation [54] to calculate the activation energy (*E*_a_) of the PEO_114_-*b*-PDM_15_-dTFSI/LiTFSI hybrids. It was found that the data at different salt doping ratios followed the VTF equation well in the experimental temperature range, and the *E*_a_ values were 8.72, 8.39, and 8.48 kJ/mol at *r* = 1/10, 1/5, and 1/3, respectively (Table S3), indicating the lowest energy barrier for the transportation of lithium ions at *r* = 1/5. 

**Figure 5:**
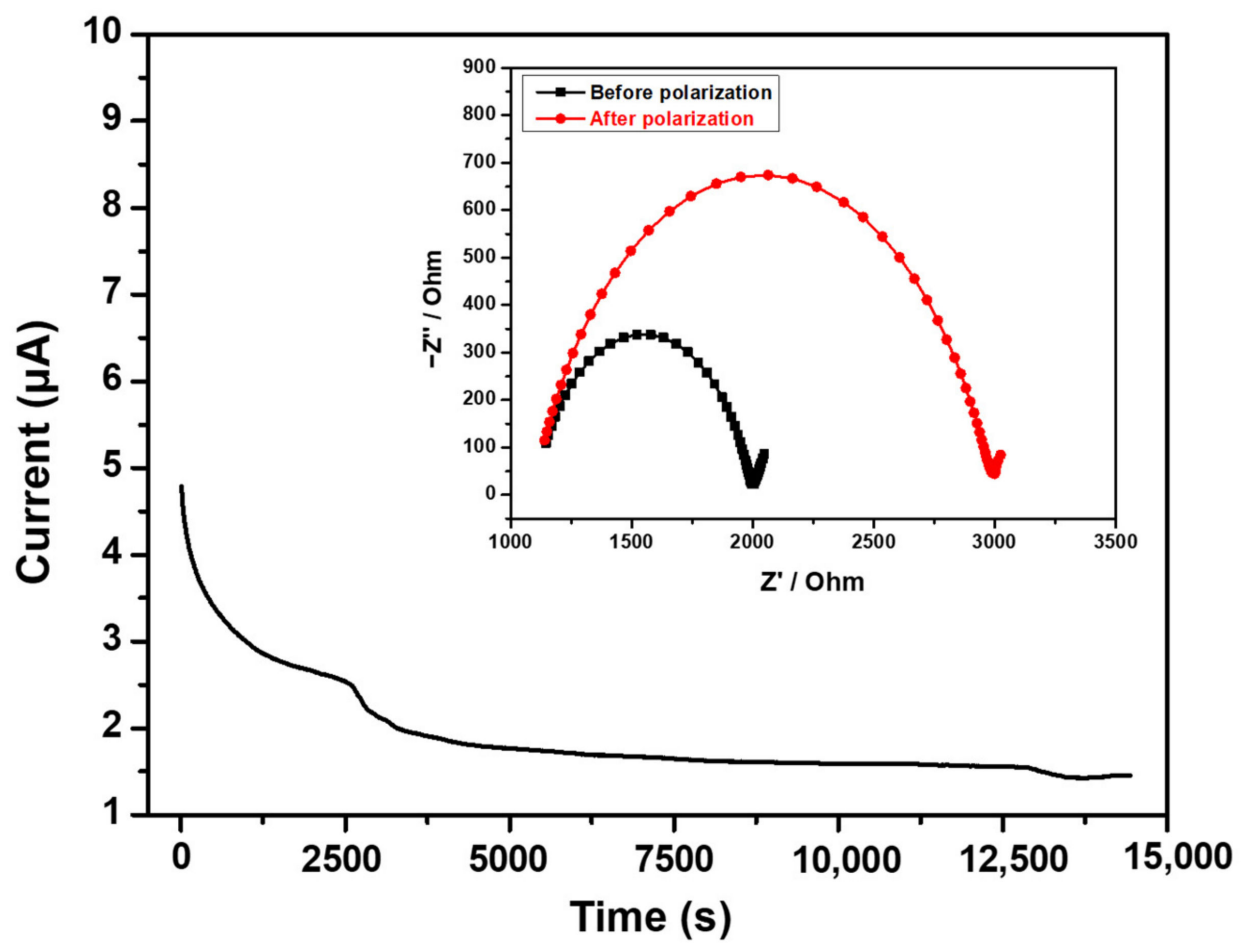
Chronoamperometry of the Li/PEO_114_-*b*-PDM_15_-dTFSI-1/5/Li cell. The inset presents the electrochemical impedance spectroscopy (EIS) before and after polarization.

The PEO_114_-*b*-PDM_15_-dTFSI-1/5 electrolyte with the highest ionic conductivity was selected to further investigate other electrochemical properties. The Li^+^ transfer number (*t*_Li_^+^) represents the migrating characteristic of lithium ions in the solid electrolytes. A large *t*_Li_^+^ value can alleviate the concentration polarization and reduce dendrite nucleation [6]. The *t*_Li_^+^ was determined by the chronoamperometric current and the impedance spectra before and after polarization (Figure 5). The calculated *t*_Li_^+^ value was 0.247 for the PEO_114_-*b*-PDM_15_-dTFSI-1/5 hybrid at 30 °C, which was slightly higher than that of the traditional PEO-based BCPs and brush polymer electrolytes [55,56,57]. A relative higher lithium ion transfer number was also achieved in some PIL/salt electrolytes compared to the neutral polymer/salt hybrid [45], and the improvement was more profound when the PILs contained multiple cations [46]. Additionally, a slightly higher *t*_Li_^+^ of 0.255 was observed in the PEO_114_-*b*-PDM_36_-dTFSI-1/5 electrolyte containing more composition of the PDM-dTFSI block (Figure S7) compared with the PEO_114_-*b*-PDM_15_-dTFSI-1/5 electrolyte. This demonstrates that the introduction of the di-cationic groups into BCPs is indeed an effective strategy to enhance *t*_Li_^+^. Due to the attractive interaction between the double quaternary ammonium cations of the charged PDM-dTFSI block and the TFSI^−^ anions, the movement of TFSI^−^ will be restricted to some extent, making for the easier migration of Li^+^.

**Figure 6:**
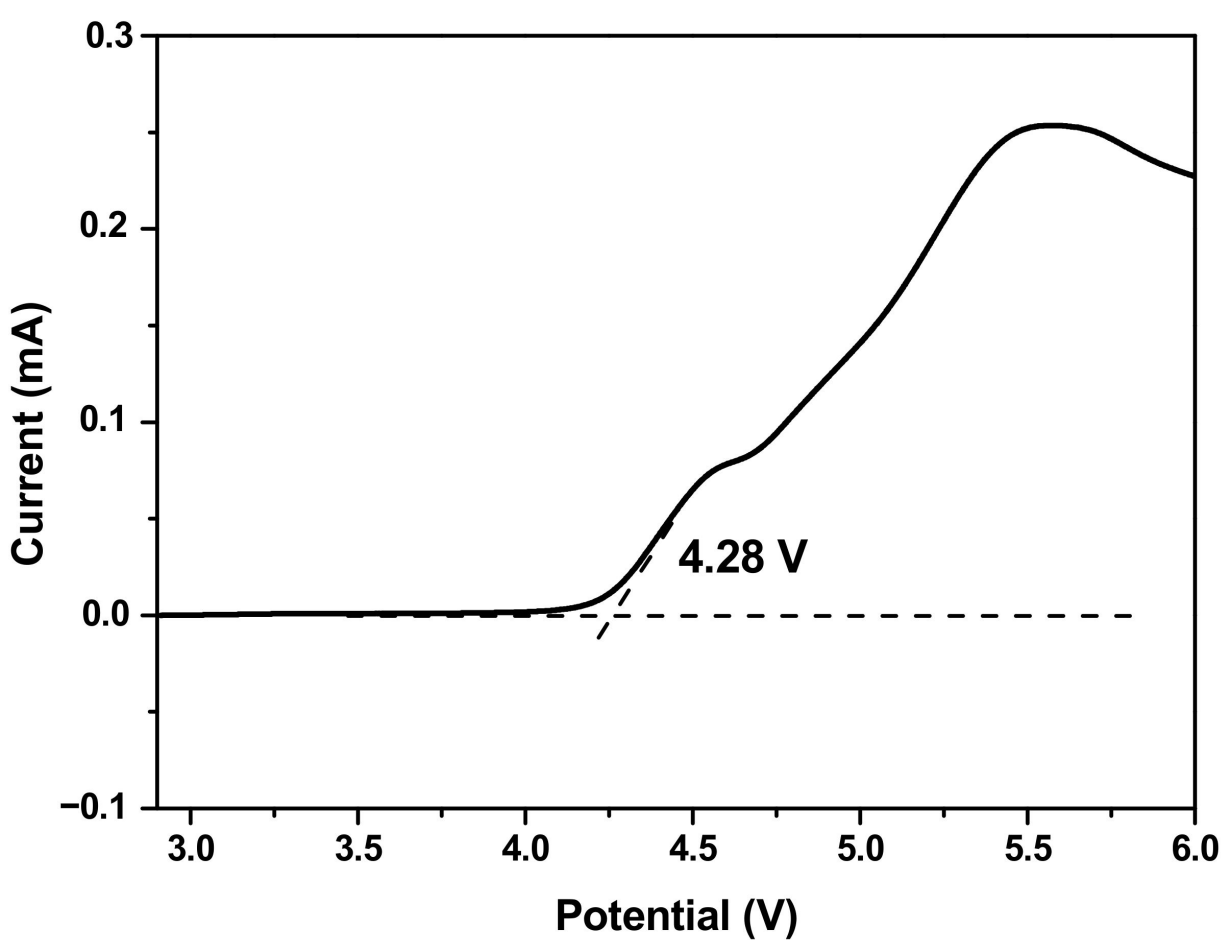
Linear sweep voltammetry (LSV) curve at a scan rate of 1 mV/s for the PEO_114_-*b*-PDM_15_-dTFSI-1/5 at 60 °C.

The electrochemical stability window of the PEO_114_-*b*-PDM_15_-dTFSI-1/5 hybrid was tested by the linear sweep voltage (LSV) at a scan rate of 1 mV/s. An oxidative decomposition voltage of ~4.28 V was observed for the PEO_114_-*b*-PDM_15_-dTFSI-1/5 hybrid (Figure 6), which was larger than the 3.8 V of the PEO/LiTFSI electrolyte [58]. This shows that the PEO_114_-*b*-PDM_15_-dTFSI-1/5 could satisfy the operation voltage when applied in Li/lithium iron phosphate (LFP) batteries.

**Figure 7:**
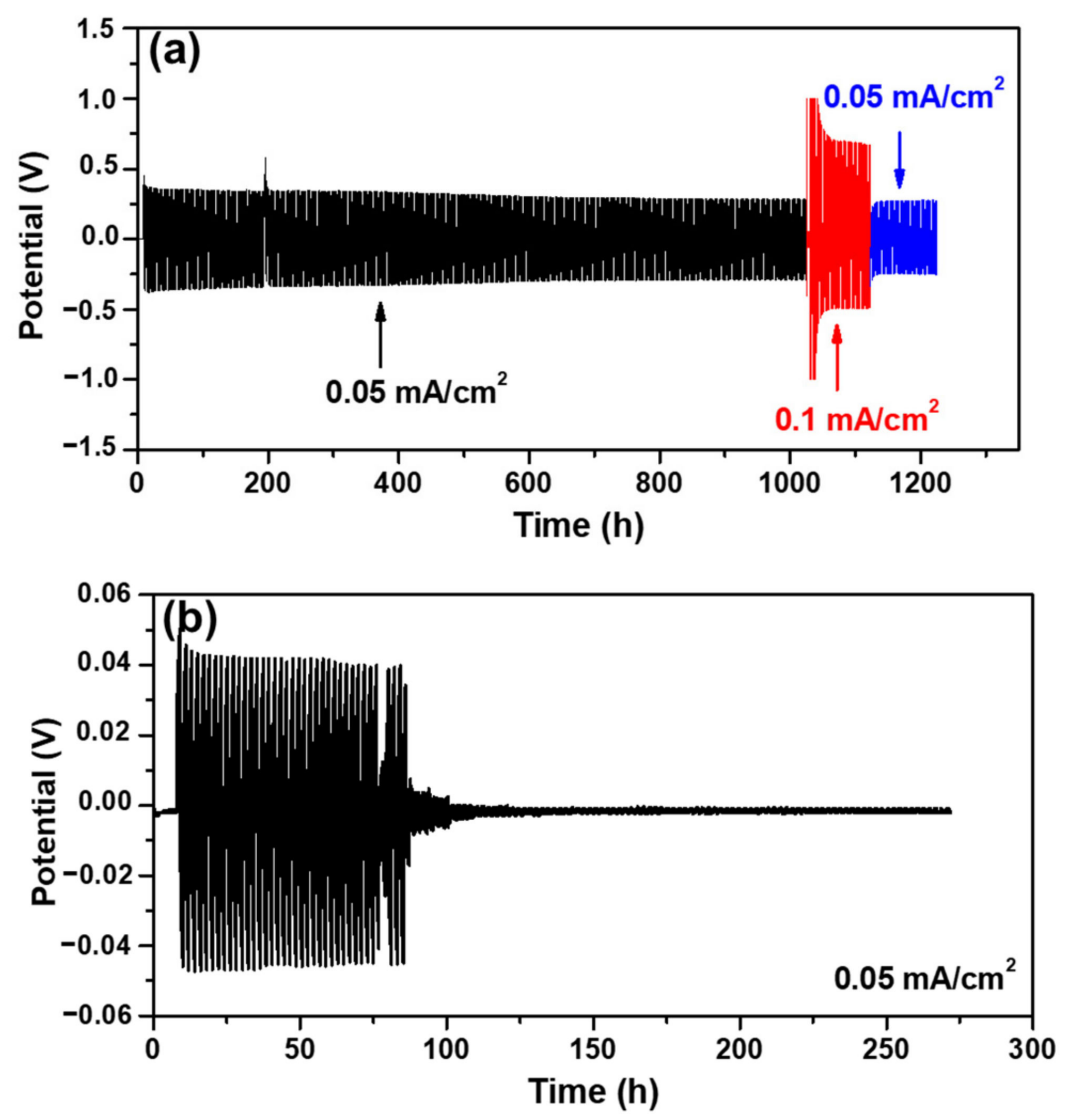
Galvanostatic cycling profiles for the Li|Li symmetrical cell with (**a**) PEO-*b*-PDM_15_-dTFSI-1/5 and (**b**) PEO/LiTFSI-1/5 electrolytes at 60 °C.

The long-term cycle property is one of the most critical indicators to measure the quality of lithium metal batteries, which represents the stability of the interface between the solid electrolyte and the electrode and the ability to inhibit lithium dendrites [59]. Therefore, galvanostatic Li plating and stripping experiments were performed on the Li/PEO_114_-*b*-PDM_15_-dTFSI-1/5/Li symmetric cell to investigate the cycling stability. Initially, the Li/PEO_114_-*b*-PDM_15_-dTFSI-1/5/Li cell was charged and discharged, respectively, for 1 h with the current density of 0.05 mA/cm^2^ at 60 °C for each cycle. As shown in Figure 7a, the symmetric cell containing the PEO_114_-*b*-PDM_15_-dTFSI-1/5 electrolyte had a small polarization voltage (~0.28 V) and no short circuit occurred after stable cycling for even 1000 h. Then, the current density was increased to 0.1 mA/cm^2^ and then suddenly decreased to 0.05 mA/cm^2^, proceeding 100 h under these two conditions, respectively. After cycling under the alternative current, the polarization voltage of the cell could still return to a stable plateau of ~0.27 V. This indicates that the solid PEO_114_-*b*-PDM_15_-dTFSI-1/5 electrolyte considerably promoted the stable and homogenous lithium stripping/plating behavior and effectively restrained the lithium dendrite during cycling, which can be attributed to the good mechanical strength of the PEO_114_-*b*-PDM_15_-dTFSI-1/5 electrolyte (Figure 3a). As a comparison, we also investigated the cycling performance of the symmetric cell with the PEO/LiTFSI hybrid. It was found that the polarization voltage became irregular after cycling for 75 h and the short circuit suddenly emerged at 100 h (Figure 7b), which was similar to the phenomena reported by other groups [60], implying the non-uniform lithium deposition and poor mechanical properties of the PEO/LiTFSI electrolyte. 

Therefore, due to the existence of the double conductive phases and the thermally stable ordered phase structure, the solid PEO_114_-*b*-PDM-dTFSI/LiTFSI electrolytes possess good ionic conductivity, Li^+^ mobility, and good mechanical strength in a wide temperature range, leading to a considerable improvement of the long-term cycling performance.

## 4. Conclusions

In conclusion, the LiTFSI-doped SBCPE with the amorphous PEO block and the charged PDM-dTFSI block were successfully developed. The PEO_114_-*b*-PDM*_n_*-dTFSI/LiTFSI electrolytes could be phase-separated into the DIS and LAM structure, depending on the composition of the BCP and doping ratio *r*. The rheology results revealed that the mechanical strength of the electrolytes was obviously enhanced due to the presence of the hard PDM-dTFSI block and the formation of the ordered and temperature-stable LAM structure, which was around 10^4^ times higher than that of the PEO/LiTFSI hybrid. In addition, LiTFSI was located in both the PEO and PDM-dTFSI domains, constructing a dual-conducting channel. After optimizing the composition and salt content, the ionic conductivity of the PEO_114_-*b*-PDM_15_-dTFSI-1/5 electrolyte with a LAM structure reached a comparably good value of 1.1 × 10^−4^ S/cm at 60 °C. This alleviates the problem of simultaneous improvement in the conductivity and mechanical strength of the solid BCP electrolyte. On the other hand, the double quaternary ammonium cations of the PDM-dTFSI block can interact with the TFSI anions, thereby accelerating the Li^+^ migration. In combination with the good mechanical and electrochemical properties, the assembled symmetric Li/PEO_114_-*b*-PDM_15_-dTFSI-1/5/Li cell can smoothly proceed for more than 1000 h, implying the stable interface between the solid electrolyte and the electrode. Therefore, this work demonstrates that the BCP electrolytes with both soft and relative rigid double conductive phases may offer new ideas for designing advanced SBCPEs used in lithium-ion batteries, for instance, taking PEO as a constituent block, we only need to explore other rigid conductive blocks that have good electrochemical properties and are incompatible with the PEO block. 

## Figures and Tables

**Table 1:** Molecular characteristics of the PEO_114_-*b*-PDM*_n_*-dTFSI samples.

Samples	*M*_n_*^a^* (g/mol)	*Ɖ ^b^*	*f*_PEO_*^c^* (%)
PEO_114_-*b*-PDM_15_-dTFSI	17,200	1.24	33.9
PEO_114_-*b*-PDM_36_-dTFSI	34,400	1.21	17.6
PEO_114_-*b*-PDM_55_-dTFSI	49,900	1.19	12.3

*^a^* Number-average molecular weight, obtained by ^1^H-NMR spectra. *^b^ Ɖ* = *M*_w_*/M*_n_, dispersity for the uncharged sample tested by GPC. *^c^* Volume fraction of PEO, calculated according to the densities of PEO and PDM-dTFSI (Table S2).

**Table 2:** Thermal properties of the PEO_114_-*b*-PDM*_n_*-dTFSI/LiTFSI hybrids.

Samples	*f*_PEO/LiTFSI_*^a^* (%)	*T*_g_^PEO^ (°C)	*T*_g_^PDM-dTFSI^ (°C)	*T*_m_^PEO^ (°C)
PEO_114_-*b*-PDM_15_-dTFSI	33.9	n/a	n/a	49.7
PEO_114_-*b*-PDM_15_-dTFSI-1/10	40.3	−36.8	74.2	n/a
PEO_114_-*b*-PDM_15_-dTFSI-1/5	45.3	−31.7	67.1	n/a
PEO_114_-*b*-PDM_15_-dTFSI-1/3	50.6	−19.3	15.7	n/a
PEO_114_-*b*-PDM_36_-dTFSI	17.6	11.5		n/a
PEO_114_-*b*-PDM_36_-dTFSI-1/10	21.9	−19.8	33.5	n/a
PEO_114_-*b*-PDM_36_-dTFSI-1/5	25.7	−15.1	23.3	n/a
PEO_114_-*b*-PDM_36_-dTFSI-1/3	29.9	−3.9		n/a

*^a^* Volume fraction of thee PEO/LiTFSI hybrid, calculated according to the densities of the PEO/LiTFSI and PDM-dTFSI/LiTFSI blocks (Table S2).

## Data Availability

Data is contained within the article and Supplementary Materials.

## Supplementary Materials

The following supporting information can be downloaded at: https://www.mdpi.com/article/10.3390/ma15227930/s1, Figures S1, S3 and S4: ^1^H-NMR spectra of PEO_114_-*b*-PDM*_n_*, PEO_114_-*b*-PDM*_n_*-dBr and PEO_114_-*b*-PDM*_n_*-dTFSI; Figure S2: GPC traces of PEO_114_-*b*-PDM*_n_*; Figures S5 and S6: SAXS profiles; Figure S7: Chronoamperometry and EIS of the Li/PEO_114_-*b*-PDM_36_-dTFSI-1/5/Li cell; Scheme S1: Synthetic routes of PEO_114_-*b*-PDM*_n_* and PEO_114_-*b*-PDM*_n_*-dTFSI; Table S1: Molecular characteristics of PEO_114_-*b*-PDM*_n_*; Table S2: Densities of PEO/LiTFSI and PDM-dTFSI/LiTFSI hybrids; Table S3: Vogel-Tammann-Fulcher (VTF) equation fitting results; Ref. [61] can be found in Supplementary Materials.
