# Supplementary material for: PEO-Based Block Copolymer Electrolytes Containing Double Conductive Phases with Improved Mechanical and Electrochemical Properties
Source: Materials (Basel). 2022 Nov 9;15(22):7930. doi: 10.3390/ma15227930 (PMC9699265; doi:10.3390/ma15227930)
Supplement: Supplementary file 1 [file materials-15-07930-s001.zip › materials-2002858-supplementary.pdf]

Supplementary Material

PEO-Based Block Copolymer Electrolytes Containing Double Conductive Phases with Improved Mechanical and Electrochemical Properties

Ze-Kun Zhang, Shi-Peng Ding, Ze Ye, Ding-Li Xia and Jun-Ting Xu *

State Key Laboratory of Motor Vehicle Biofuel Technology, International Research Center for X Polymers, Department of Polymer Science and Engineering, Zhejiang University, Hangzhou 310027, China;

*Correspondence: xujt@zju.edu.cn

Contents:

1. Part 1: Synthetic routes and structure characterization: **S2**
2. Part 2: Densities of PEO/LiTFSI and PDM-dTFSI/LiTFSI hybrids: **S7**
3. Part 3: SAXS profiles: **S8**
4. Part 4: Vogel−Tammann−Fulcher (VTF) equation fitting results for the experimental ionic conductivity: **S9**
5. Part 5: Measurement of lithium ion transfer number: **S9**

Part 1: Synthetic routes and structure characterization

Synthesis of PEO_{114}-*b*-PDM_n BCPs

The PEO-*b*-PDM BCPs were synthesized by atom transfer radical polymerization (ATRP). Firstly, the PEO_{114}-Br macroinitiator was prepared by esterification of PEO and 2-bromoisobutyryl bromide. Then, a series of PEO_{114}-*b*-PDM_n BCPs were obtained through conducting the ATRP polymerization of DM, where n is the polymerization degree of PDM block. The specific polymerization process is shown in Scheme S1. Taking PEO_{114}-*b*-PDM_{15} as an example, PEO_{114}-Br (3 g, 0.6 mmol), DM (3 mL, 18 mmol), and dry THF (27.0 mL) were charged into a Schlenk flask under nitrogen atmosphere and stirred for 3 min. The reaction mixture was degassed by three freeze-pump-thaw cycles. The HMTTA (0.65 mL, 2.40 mmol) and CuBr (129 mg, 0.9 mmol) was added under nitrogen atmosphere at the second thaw and last freeze state, respectively. The reaction mixture was immerged in to the oil bath, stirring at 65 °C for 3 hours. The polymerization was terminated by quenching the reactants with liquid nitrogen. The mixture was diluted with THF and then passed through a neutral alumina column using THF as the eluent to remove the metal complex. The solution was concentrated and precipitated in cold *n*-hexane twice. Finally, the final product was dried in a vacuum oven at 60 °C for 24 h. The polymerization degree of PDM was calculated by the area ratio of peak b and a in the ^{1}H-NMR spectra shown in Figure S1.The number-average molecular weight and polydispersity index are shown in Figure S2 and Table S1

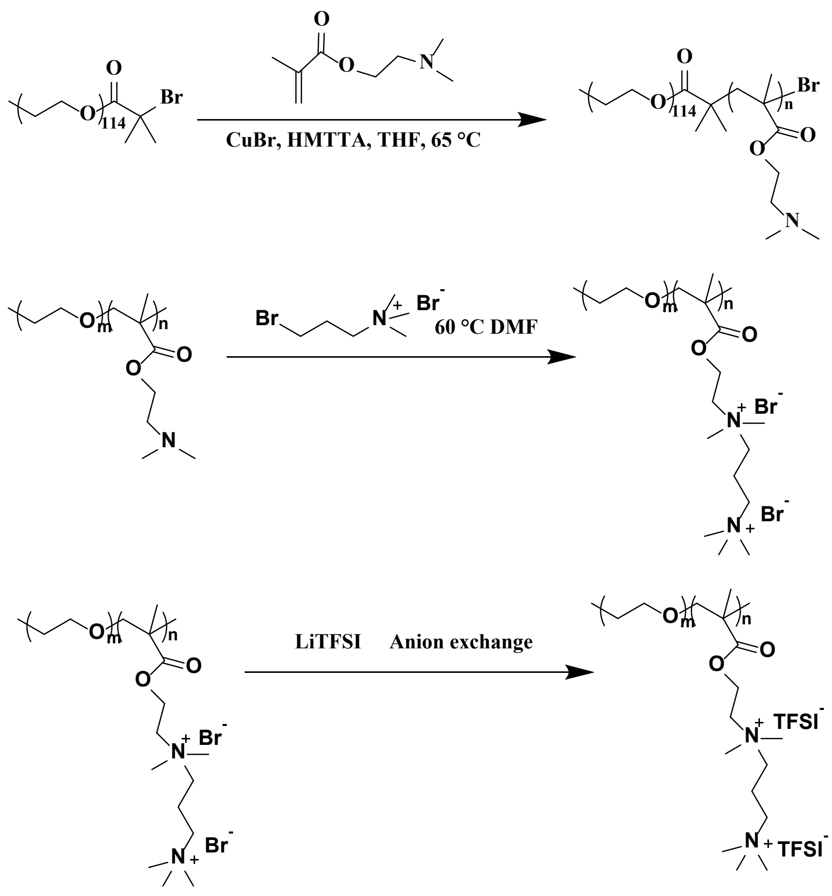

Scheme. S1. The synthetic routes of PEO_{114}-*b*-PDM_n and PEO_{114}-*b*-PDM_n-dTFSI.

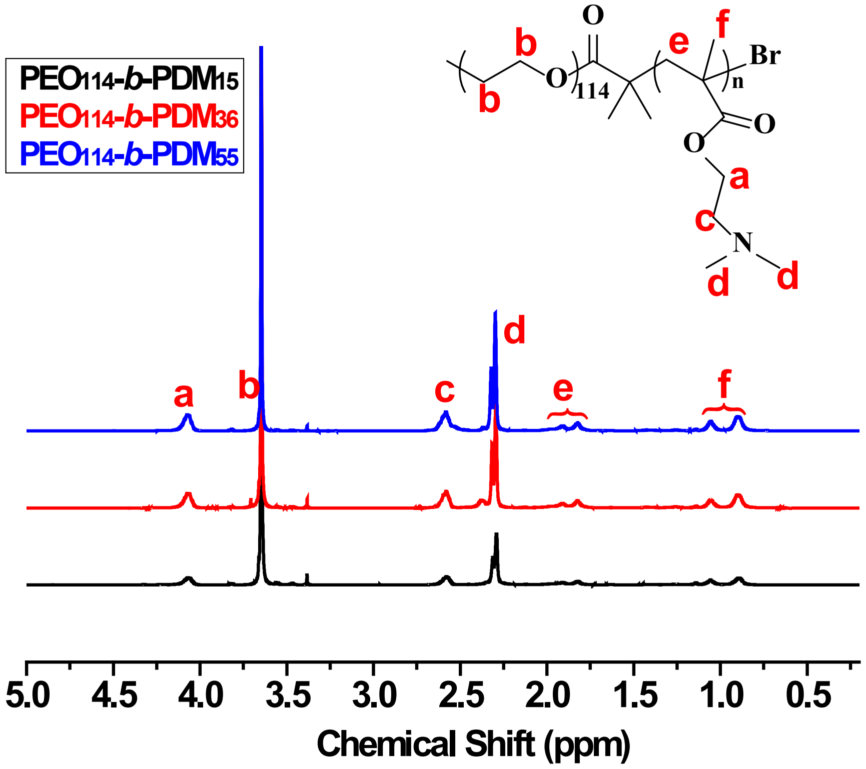

Figure. S1. ^{1}H-NMR spectra of PEO_{114}-*b*-PDM_n.

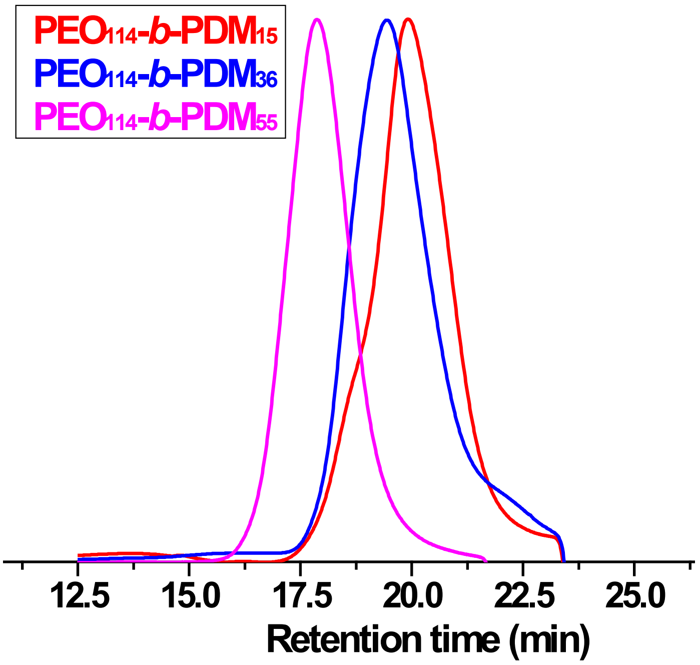

Figure. S2. GPC traces of PEO_{114}-*b*-PDM_n.

Table S1. Molecular characteristics of PEO_{114}-*b*-PDM_n

Samples	$M_n{}^a$ (g/mol)	$Đ^b$
PEO_{114}-*b*-PDM_{15}	7400	1.24
PEO_{114}-*b*-PDM_{36}	10700	1.21
PEO_{114}-*b*-PDM_{55}	13600	1.19

^{a}Determined from the ^{1}H-NMR spectra. $^bĐ = M_w/M_n$, polydispersity index of the sample determined by GPC.

Synthesis of PEO_{114}-*b*-PDM_n-dTFSI BCPs

To ensure the full quaternization, the feed ratio of (3-bromopropyl)trimethylammonium bromide to the PDM unit of PEO_{114}-*b*-PDM_n block was set to 10:1. Typically, as shown in the process of Scheme S1, PEO_{114}-*b*-PDM_{15} (3.2 g, 0.43 mmol) and (3-bromopropyl)trimethylammonium bromide (16.8 g, 64.5 mmol) were dissolved in around 100 mL DMF in the schlenk flask, and then the flask was immersed in an oil bath at 60 °C under a nitrogen atmosphere. After stirring for 48 hours, the solution was directly dialyzed against DMF (three days) and deionized water (one day). The final product was vacuum freeze-dried to obtain the PEO_{114}-*b*-PDM_{15}-dBr.The conversion of the quaternization is > 99%, which is calculated by the ^{1}H-NMR as shown in Figure. S3, " f " versus resonance " b " (i.e., f / b) = 1.0). The PEO_{114}-*b*-PDM_{15}-dTFSI were obtained by anion exchange reaction on PEO_{114}-*b*-PDM_{15}-dBr. PEO_{114}-*b*-PDM_{15}-dBr (1 g, 0.09 mmol) and LiTFSI (1.15 g, 3.99 mmol, weighed in a glove box) were dissolved in 3 mL and 5 mL DMF, respectively, and then mix them in a 25 mL round bottom flask. After the mixture was stirred at 60 °C for 48 hours, it was dialyzed against DMF (3 days) and deionized water (2 days) to directly remove the excess salts from the solution. The obtained polymer was freeze-dried under vacuum to obtain a pale yellow powder. The NMR results of PEO_{114}-*b*-PDM_n-dTFSI BCPs are shown in Figure S4.

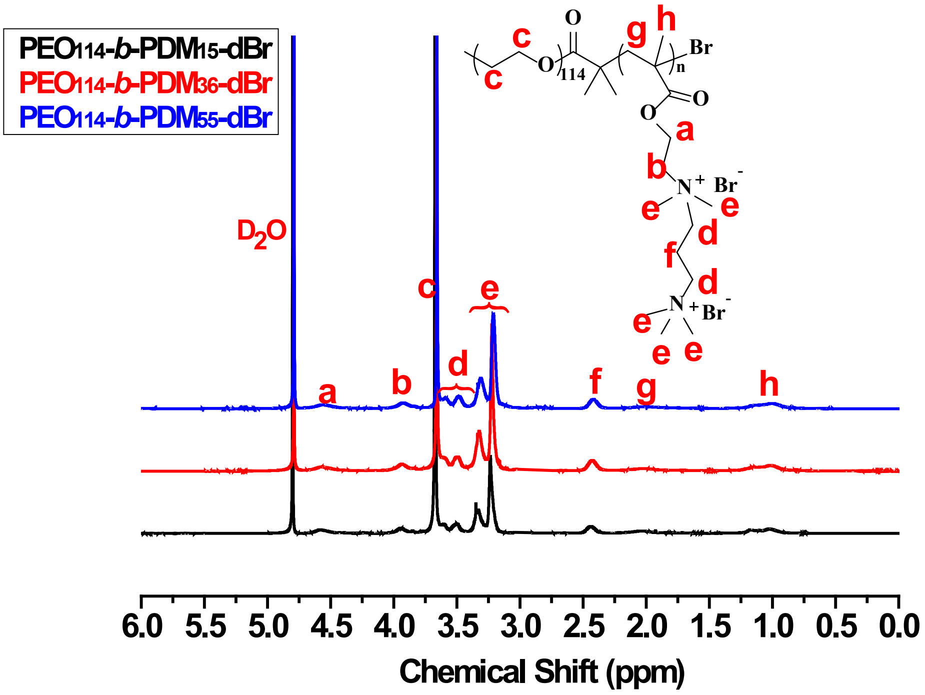

Figure. S3. ^{1}H-NMR spectra of PEO$_{114}$-*b*-PDM$_n$-dBr.

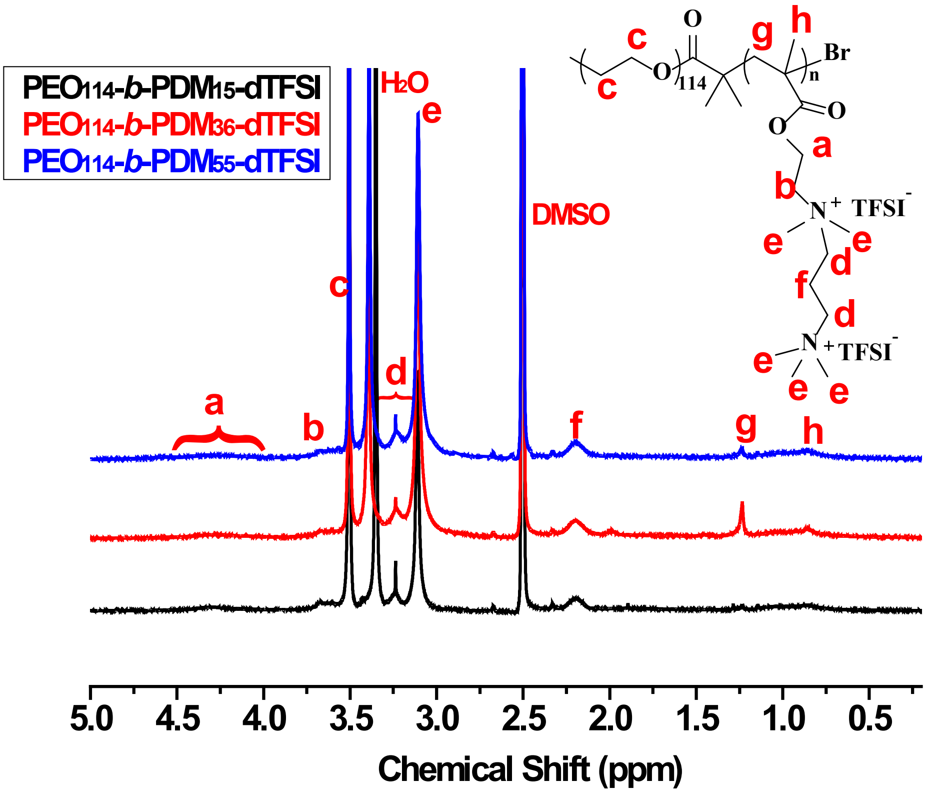

Figure. S4. ^{1}H-NMR spectra of PEO$_{114}$-*b*-PDM$_n$-dTFSI.

Part 2: Densities of PEO/LiTFSI and PDM-dTFSI/LiTFSI hybrids

The densities of PEO/LiTFSI and PDM-dTFSI/LiTFSI hybrids are calculated as the following formulas:

$$\rho_{\text{PEO/Salt}} = \frac{1}{\frac{w_{\text{PEO}}}{\rho_{\text{PEO}}} + \frac{w_{\text{Salt}}}{\rho_{\text{Salt}}}} \quad \text{(S1)}$$

$$\rho_{\text{PDM-dTFSI/Salt}} = \frac{1}{\frac{w_{\text{PDM-dTFSI}}}{\rho_{\text{PDM-dTFSI}}} + \frac{w_{\text{Salt}}}{\rho_{\text{Salt}}}} \quad \text{(S2)}$$

where w_{PEO}, $w_{\text{PDM-dTFSI}}$ and w_{Sallt} are the weight percentages of the PEO, PDM-dTFSI and lithium salt (LiTFSI), ρ_{PEO}, $\rho_{\text{PDM-dTFSI}}$ and ρ_{Salt} are the densities of PEO, PDM-dTFSI and LiTFSI. The densities used for calculation are 1.06, 1.34 and 2.02 g/ cm^3, for PEO, PDM-dTFSI and LiTFSI, respectively [1]. The density of PDM-dTFSI was measured by the Archimedes Method [2].

Table S2. Densities of PEO/LiTFSI and PDM-dTFSI/LiTFSI hybrids with the doping ratio r = 1/10, 1/5 and 1/3

Samples	Density (g/cm^3)
PEO	1.06
PEO-1/10	1.31
PEO-1/5	1..45
PEO-1/3	1.57
PDM-dTFSI	1.34
PDM-dTFSI-1/10	1.36
PDM-dTFSI-1/5	1.37
PDM-dTFSI-1/3	1.39

Part 3: SAXS profiles

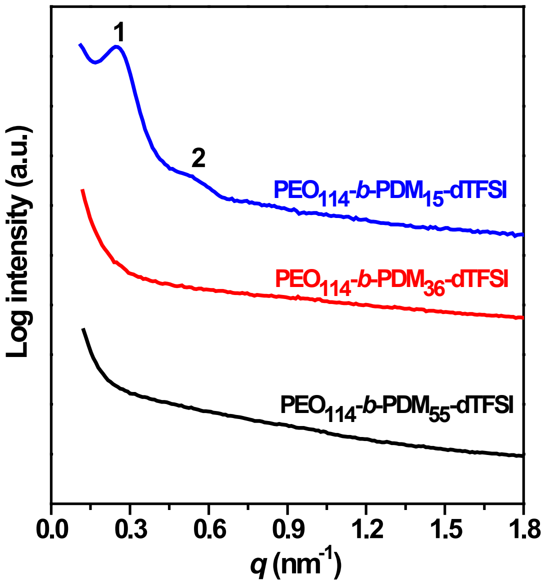

Figure S5. SAXS profiles of the PEO_{114}-*b*-PDM_n-dTFSI BCPs at 30 °C.

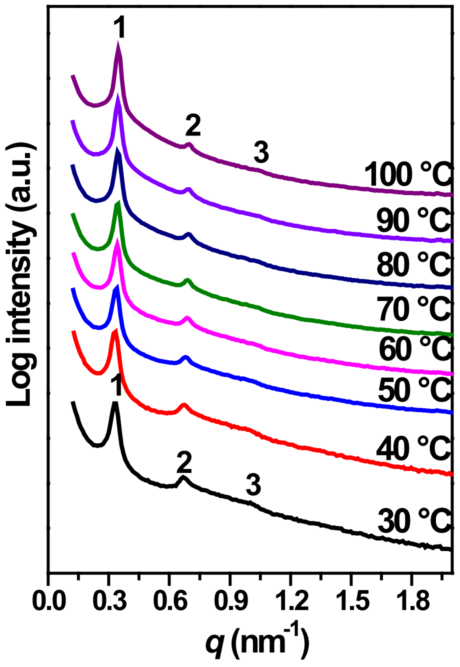

Figure S6. Temperature-variable SAXS profiles of the PEO_{114}-*b*-PDM_{15}-dTFSI/LiTFSI electrolyte at r = 1/10.

Part 4: The Vogel-Tammann-Fulcher (VTF) equation fitting results for the experimental ionic conductivity

Table S3. The Vogel-Tammann-Fulcher (VTF) equation ($\ln \sigma = -E_a/R(T-T_0) + \ln A$) fitting results for the experimental ionic conductivity (σ) of the PEO_{114}-*b*-PDM_{15}-dTFSI/LiTFSI hybrids, where E_a is the activation energy value for the transportation of lithium ions, T_0 is the Vogel temperature [3].

Doping ratio *r*	ln*A*	E_a (kJ/mol)	R^2
1/10	-2.60	8.72	0.998
1/5	-1.97	8.39	0.999
1/3	-1.37	8.48	0.990

Part 5: Measurement of lithium ion transfer number

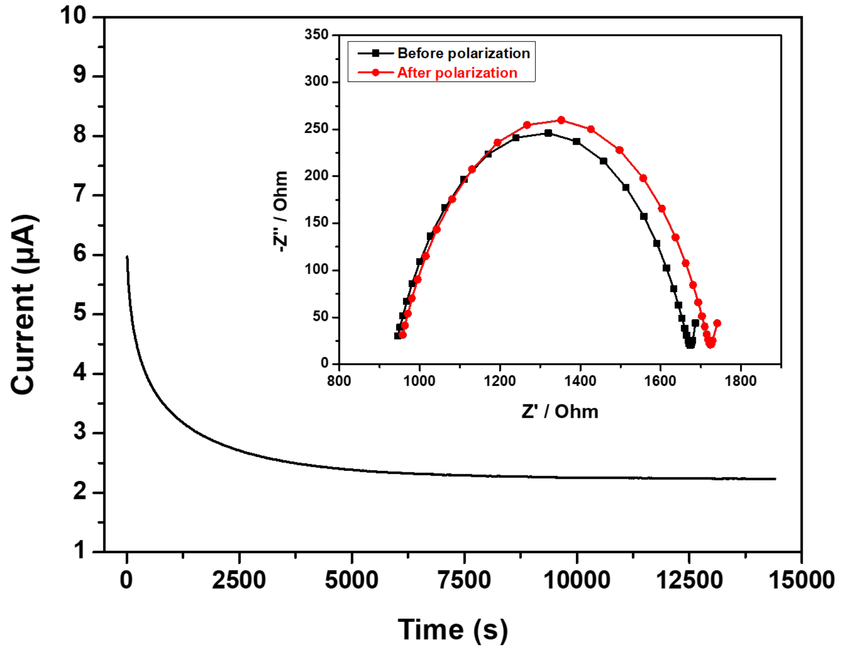

Figure S7. Chronoamperometry of the Li/PEO_{114}-*b*-PDM_{36}-dTFSI-1/5/Li cell. The inset presents the electrochemical impedance spectroscopy (EIS) before and after polarization.

References

1. Cao, X. H.; Yang, J. L.; Wang, R. Y.; Zhang, X. H.; Xu, J. T. Microphase Separation of Poly(propylene monothiocarbonate)-*b*-poly(ethylene oxide) Block Copolymers Induced by Differential Interactions with Salt. *Polymer* **2019**, *180,* 121745.

2. Zhang, Z. K.; Ding, S. P.; Ye, Z.; Xia, D. L.; Xu, J. T. Thermodynamic Understanding the Phase Behavior of Fully Quaternized Poly(ethylene oxide)-B-Poly(4-Vinylpyridine) Block Copolymers. *Polymer* **2022**, *254,* 125045.

3. Diederichsen, K. M.; Buss, H. G.; McCloskey, B. D. The Compensation Effect in the Vogel–Tammann–Fulcher (VTF) Equation for Polymer-Based Electrolytes. *Macromolecules* **2017**, *50,* 3831-3840.
